# The influence of explainable vs non-explainable clinical decision support systems on rapid triage decisions: a mixed methods study

**DOI:** 10.1186/s12916-023-03068-2

**Published:** 2023-09-19

**Authors:** Daniel Laxar, Magdalena Eitenberger, Mathias Maleczek, Alexandra Kaider, Fabian Peter Hammerle, Oliver Kimberger

**Affiliations:** 1 Department of Anaesthesia, Intensive Care Medicine and Pain Medicine, Medical University of Vienna, Vienna, Austria; 2 Ludwig Boltzmann Institute Digital Health and Patient Safety, Ludwig Boltzmann Gesellschaft, Vienna, Austria; 3 Center for Medical Data Science, Medical University of Vienna, Vienna, Austria

**Keywords:** Triage, Decision process, Clinical decision support systems, Machine learning, Human–computer interaction

## Abstract

**Background:**

During the COVID-19 pandemic, a variety of clinical decision support systems (CDSS) were developed to aid patient triage. However, research focusing on the interaction between decision support systems and human experts is lacking.

**Methods:**

Thirty-two physicians were recruited to rate the survival probability of 59 critically ill patients by means of chart review. Subsequently, one of two artificial intelligence systems advised the physician of a computed survival probability. However, only one of these systems explained the reasons behind its decision-making. In the third step, physicians reviewed the chart once again to determine the final survival probability rating. We hypothesized that an explaining system would exhibit a higher impact on the physicians’ second rating (i.e., higher weight-on-advice).

**Results:**

The survival probability rating given by the physician after receiving advice from the clinical decision support system was a median of 4 percentage points closer to the advice than the initial rating. Weight-on-advice was not significantly different (*p* = 0.115) between the two systems (with vs without explanation for its decision). Additionally, weight-on-advice showed no difference according to time of day or between board-qualified and not yet board-qualified physicians. Self-reported post-experiment overall trust was awarded a median of 4 out of 10 points. When asked after the conclusion of the experiment, overall trust was 5.5/10 (non-explaining median 4 (IQR 3.5–5.5), explaining median 7 (IQR 5.5–7.5), *p* = 0.007).

**Conclusions:**

Although overall trust in the models was low, the median (IQR) weight-on-advice was high (0.33 (0.0–0.56)) and in line with published literature on expert advice. In contrast to the hypothesis, weight-on-advice was comparable between the explaining and non-explaining systems. In 30% of cases, weight-on-advice was 0, meaning the physician did not change their rating. The median of the remaining weight-on-advice values was 50%, suggesting that physicians either dismissed the recommendation or employed a “meeting halfway” approach. Newer technologies, such as clinical reasoning systems, may be able to augment the decision process rather than simply presenting unexplained bias.

**Supplementary Information:**

The online version contains supplementary material available at 10.1186/s12916-023-03068-2.

## Background

Decision support systems are designed to aid human decision-making processes. In healthcare, the term clinical decision support systems (CDSS) is used to refer to a system that “provides clinicians, staff, patients or other individuals with knowledge and person-specific information, intelligently filtered or presented at appropriate times, to enhance health and health care” [1]. CDSS have a wide range of applications which can include supporting therapeutic decision-making and estimating prognosis, as well as diagnostics and even triage [1, 2].

While under normal circumstances triage systems are designed to prioritize patients, during episodes of acute shortage of treatment capacities, triage may also be used to differentiate between patients who will receive curative care and those who will receive only palliative care [3]. As such, most triage systems used in mass casualty situations have a category for patients who are only expected to make a recovery if a large amount of resources is allocated to them [4]. While this is intended to achieve the best outcome for most patients, it poses an ethical dilemma [3, 5].

The outbreak of the SARS-CoV-2 pandemic spiked both public and healthcare professional interests in the process of triage [6, 7]. Numerous decision support systems have been developed, validated or modified specifically for COVID-19, many of which are based on novel machine-learning technologies and algorithms [2]. In the face of failing and overwhelmed healthcare systems, some authors have considered the use of diagnostic and prognostic models in situations requiring triage [8, 9]. While to the best of our knowledge no CDSS have been used as the primary method for triaging patients to date, advances in technology are likely to pose similar use cases in the future. Most recently, newer machine learning models have fuelled public discussion about the ethical use of these systems in medicine [10, 11]. Against the background of experiments suggesting obedient human behaviour in stressful situations influenced by robots and machines [12–14], it is vital to understand the interactions between CDSS and professional healthcare providers. Triage decisions must be made quickly, exposing the physician to a significant level of stress [15]. Therefore, we set out to examine the effects of different CDSS on healthcare providers’ decision-making processes in a simulated triage situation.

We hypothesized that critical care physicians are more likely to accept the recommendation of a CDSS which provides an explanation of the underlying decision-making process.

## Methods

This study recruited 32 physicians, all residents (*n* = 21) or staff physicians (*n* = 11) at the Department of Anaesthesia, Intensive Care Medicine, and Pain Medicine at the Medical University of Vienna. This is Austria’s largest tertiary care centres, with 134 intensive care units (ICU) beds and 59 operating theatres. The study was approved by the Medical University of Vienna ethics board (EK 2293/2020) in December 2020. Anaesthesiologists and intensivists were then recruited on a first-come first-served basis via mailing lists and department-wide messaging groups.

Briefing and supervision of the study sessions were all performed by the same person (DL). All study sessions were structured equally. First, the physician was informed about the study procedure and duration of the experiment before providing written consent. Then, the participant was given time to complete the initial questionnaire before receiving their detailed study briefing. This qualitative questionnaire, with open-ended, written answers, asked the participant about their knowledge of triage in their profession, as well as previous and current triage experience. In the subsequent study briefing, the participant was informed that 59 patients—currently admitted to normal wards—were critically ill and needed to be evaluated for intensive care admission. They were informed that “two independent artificial intelligence systems using the latest machine learning technology” were designed to aid their decision-making. However, only one system would be available to each patient. Then, they were told about the three tasks they had to complete for each patient: (i) to rate the survival probability of the patient under the premise of ICU admission and on the basis of health records provided, (ii) to re-rate the survival probability for the patient under the premise of ICU admission and based on the health records provided as well as the rating given by the decision support system, and (iii) to decide whether the patient should be admitted to the ICU or receive palliative care in view of the limited resources available due to the ongoing pandemic. The ICU resource limitations were not specified further. The participants were informed of the 4-h time limit for evaluating all 59 patients during the experiment. After evaluating all the patients, the participant was asked in a quantitative questionnaire to reflect on the factors which had played a role in their triage decisions. In February 2023, after all the experiments had been completed, the participants were asked to reflect on their experiences and the extent to which they had trusted either system to correctly estimate patient survival.

The 59 health records were derived from actual, critical patients in a ward at our centre. All patient identifiers were eliminated from these records. The paper-based patient records were presented to each physician in a randomized order. An example patient file cover is shown in Additional file 1: Fig. S1.

**Fig. 1:**
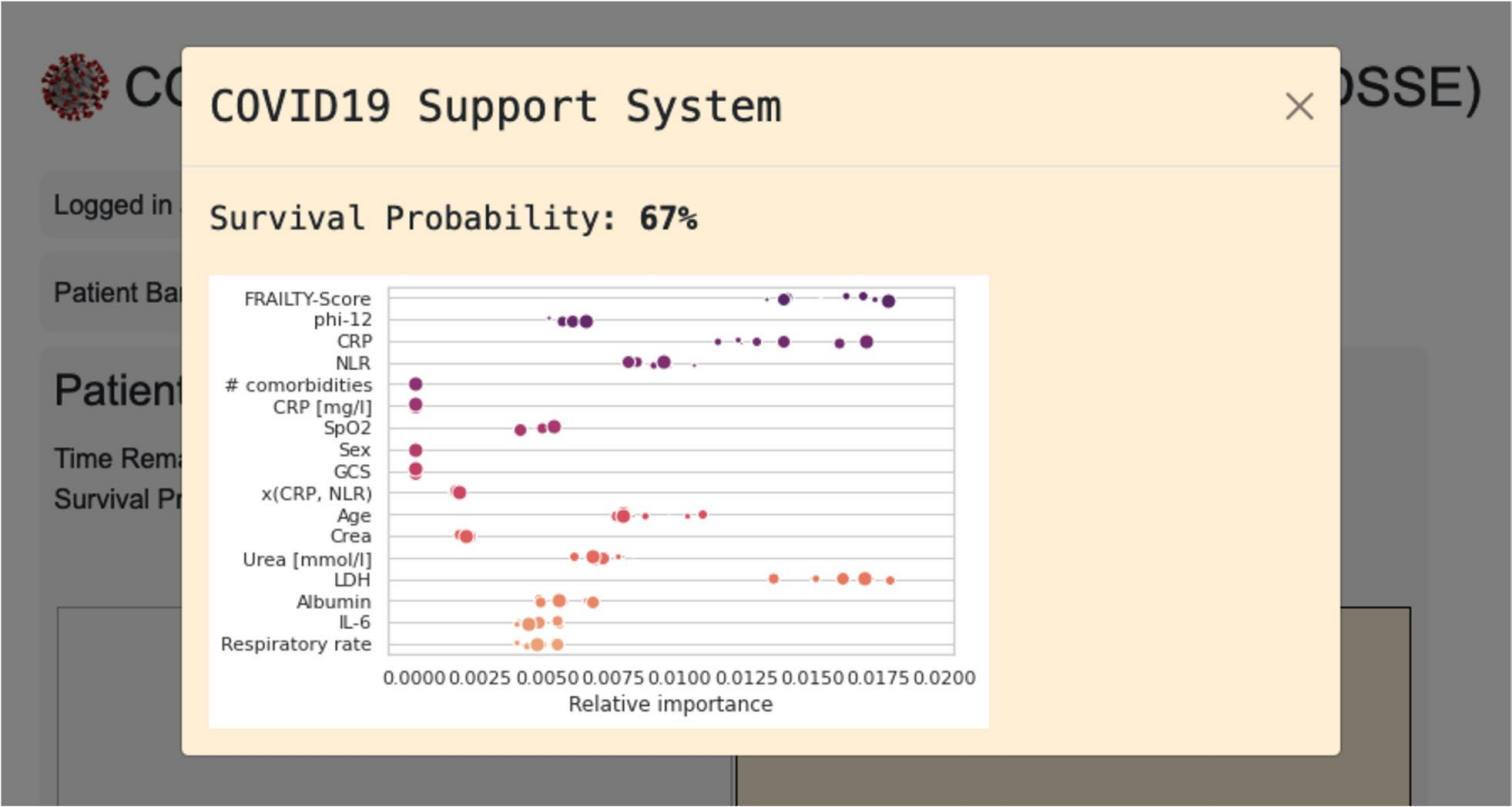
The explanation provided. This type of graphic was shown to the examiner by the explaining CDSS system. It was presented alongside the rated survival probability. The figure shows true and fictional influential factors (left) rated by their relative importance. The distribution and dot size are intended to represent some degree of variability. All values were generated randomly

While participants believed that two different decision support systems with different user interfaces had been available (one non-explaining, one explaining), both systems presented the same predefined survival probability. However, one of the interfaces provided an explanation of its decision. This was achieved by showing the most influential factors in the scoring formula which was based on the 4C score by Knight et al. [16] and is shown in Fig. 1. The system made available to each patient and physician was randomized. Details on the randomization can be found in Additional file 1: Fig. S2.

For each evaluation, we recorded the initial survival probability rating, the advice given and the subsequent rating after chart review and advice.

Weight-on-advice (WoA) was calculated as described in [17]:

$${\text{WoA}}=\frac{\text{initial rating}-\text{rating after advice}}{\text{initial rating}-{\text{advice}}}$$

WoA was limited to the range between 0 and 1, both inclusive [18]. Samples in which the WoA could not be calculated because the initial rating and advice were identical were excluded. A WoA of 0 depicts no change between the initial rating and the rating after advice, while a WoA of 1 represents full acceptance of the system’s advice.

The required sample size was calculated for 80% power and a type 1 error of 0.05 based on an estimated WoA difference of 0.2 between the two systems for a two-sided two-sample *t*-test which was the primary outcome parameter.

Normally distributed, continuous variables are reported as mean and standard deviation (SD). Non-normally distributed, continuous variables and ordinal values are reported as median and interquartile range (IQR). The correlation between influential factors and the physician’s median WoA was calculated using Spearman’s correlation coefficient.

Linear mixed models were used to compare the primary WoA outcome between the randomized groups (advice with vs without explanation) and between board-qualified physicians and residents, respectively. Furthermore, linear mixed models were calculated to evaluate a potential dependency between WoA, time of day and progress of the experiment. In addition to these fixed factors (group, board certification, time of day, progress of the experiment), the patient and physician effects were considered as random factors in order to incorporate the dependency structure of repeatedly measured observations of the same individual and by the same physician, respectively. A similar linear mixed model was calculated to analyse the influence of the progress of the experiment on the decision time (log2-transformed). Another linear mixed model was calculated, considering the two binary factors: advice (with vs without explanation) and advice at the first patient (with vs without explanation), and the interaction term of these two factors. Generalized linear mixed models were performed to analyse the binary outcome admission to the ICU using the logit link function. Again, the patient and physician effects were considered as random factors, and the explanatory variables qualification, progress of the experiment and rating after advice were considered as fixed factors and covariates. To evaluate a potential difference in the effect of the rating after advice depending on the physician’s qualification, an interaction term was tested in the model. The strengths of the effects are described by odds ratios (OR) with 95% confidence intervals (95%CI).

Statistical analyses were performed using SAS (version 9.4, SAS Institute Inc. (2020), Cary, NC, USA). An alpha value of 0.05 was set as the threshold for statistical significance.

## Results

All 32 physicians (21 residents and 11 staff physicians) completed the study procedure by evaluating 59 patients each, giving 1888 physician–patient evaluations in total. No participant dropped out of the study. Experiments were conducted between December 2021 and June 2022.

All participants adhered to the specified time limit. The median (IQR) time for completion was 179 (149–203) min, with the maximum time allowance being 240 min. On average, physicians were shown an explanation for the advice 29.8 out of 59 times (SD 3.8), and on average, an explanation was available 16.1 out of 32 times for a patient (SD 1.1).

**Fig. 2:**
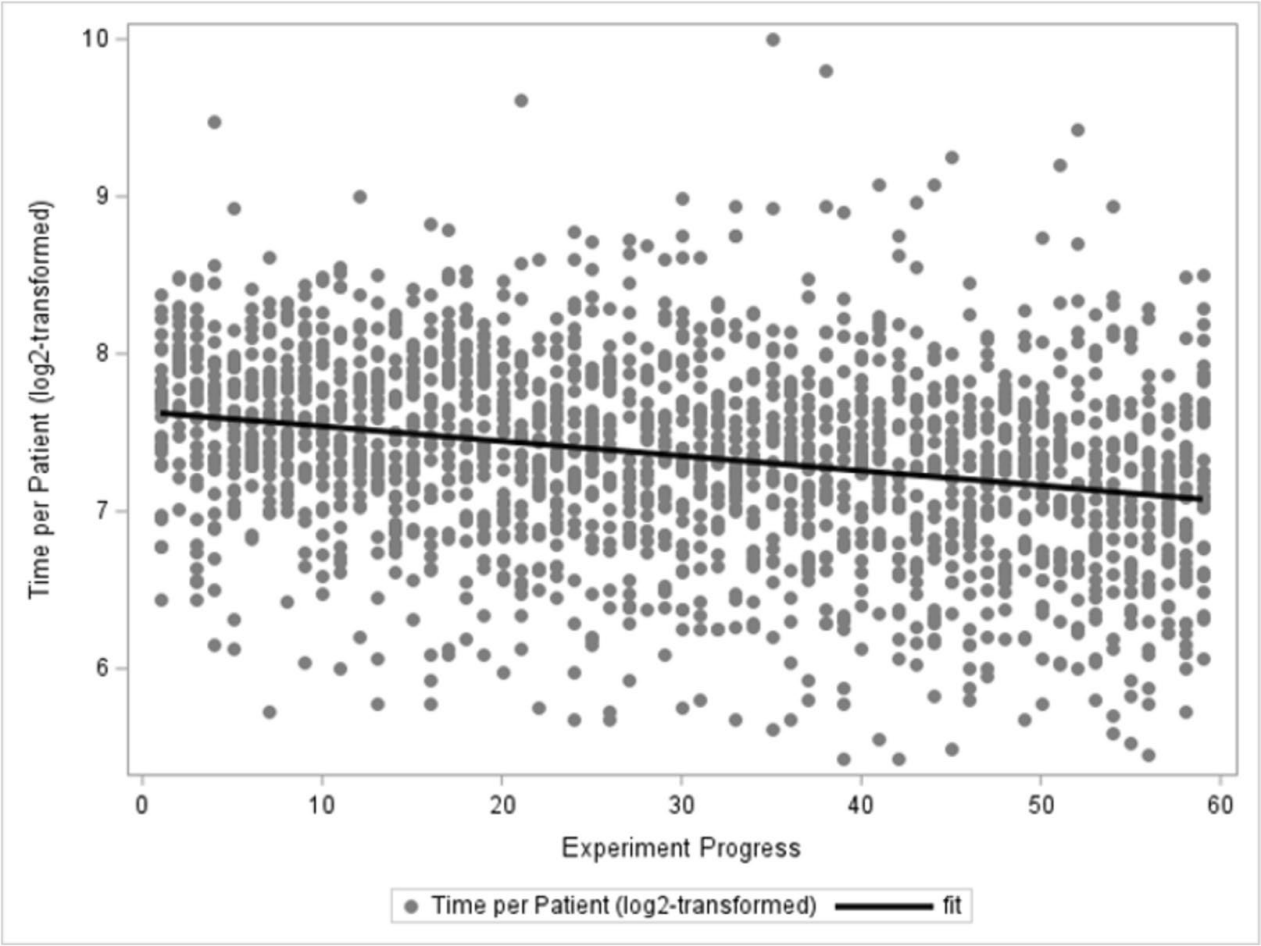
The *x*-axis shows the progress of the experiment; the *y*-axis shows the number of seconds spent on each individual patient

The median (IQR) time spent on the first patient was 198 (162–228.5) s while the median time spent on the 59th patient was 152 (120–217) s. This decline in decision time per patient was statistically significant (*p*-value < 0.0001) and is depicted in Fig. 2.

**Fig. 3:**
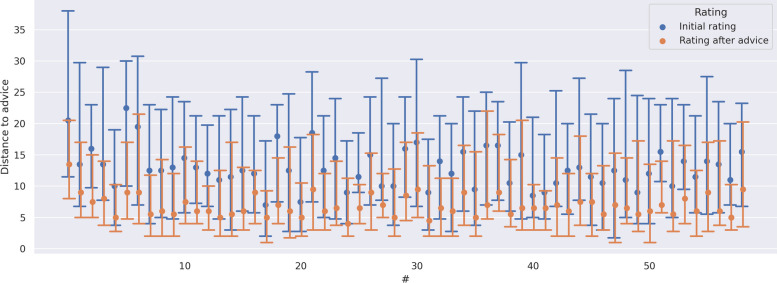
Distance of initial rating and rating after advice. The *x*-axis shows the progress of the experiment; the *y*-axis shows the median distance to the advice for each patient with IQR error bars—both the initial rating and the rating after advice are displayed. The mean change between the initial rating and the rating after advice is 5.38% absolute (33.8% relative)

**Fig. 4:**
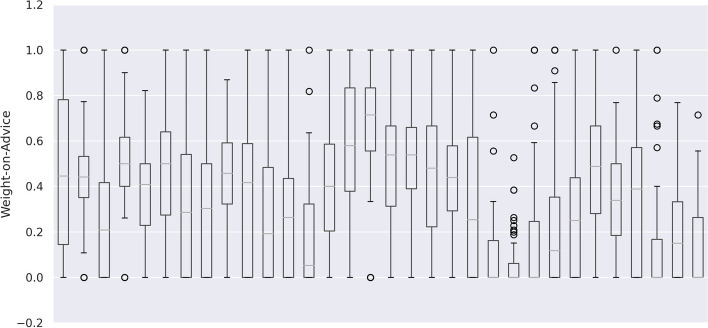
Weight-on-advice by physician. Each vertical lane represents the calculated weight-of-advice values for a single physician. Boxes contain Q1 through Q3; whiskers extend to the farthest observation within 1.5 times the IQR. Outliers are shown as circles. Weight-of-advice values were limited to between 0 and 1

WoA was calculated for both systems to determine whether providing an explanation influenced the impact of the decision support system. In 72 instances (3.8%), the physician’s initial and final survival probability rating precisely matched the rating given by the CDSS as advice. These cases were excluded from WoA calculations. The median (IQR) WoA was 0.33 (0.0–0.56). WoA was 0.35 (0.0–0.57) without explanation and 0.31 (0.0–0.54) for the system which provided an explanation (*p*-value = 0.115). This is equivalent to a median of a 4 percentage point change in the rating towards the direction of the CDSS advice (see Fig. 3). Figure 4 shows the distributions of WoA per physician, while Additional file 1: Fig. S3 shows the distribution of WoA by physician and explanation.

**Fig. 5:**
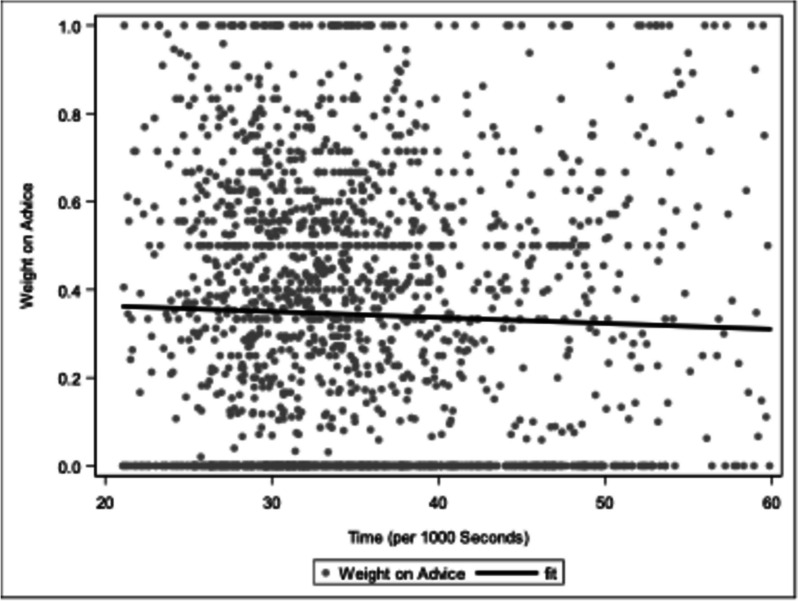
Weight-on-advice over the course of the day. No statistically significant trend can be observed

There was no difference in WoA (*p*-value = 0.33) between residents (median 0.37, IQR 0–0.56) and board-certified physicians (median 0.25, IQR 0–0.53). WoA did not change over the course of the experiment (*p*-value = 0.194). WoA did not change over the course of a day (*p*-value = 0.446), which is depicted in Fig. 5. The first interaction (with or without explanation) did not significantly influence the further course of the experiment (*p*(interaction) = 0.815).

**Fig. 6:**
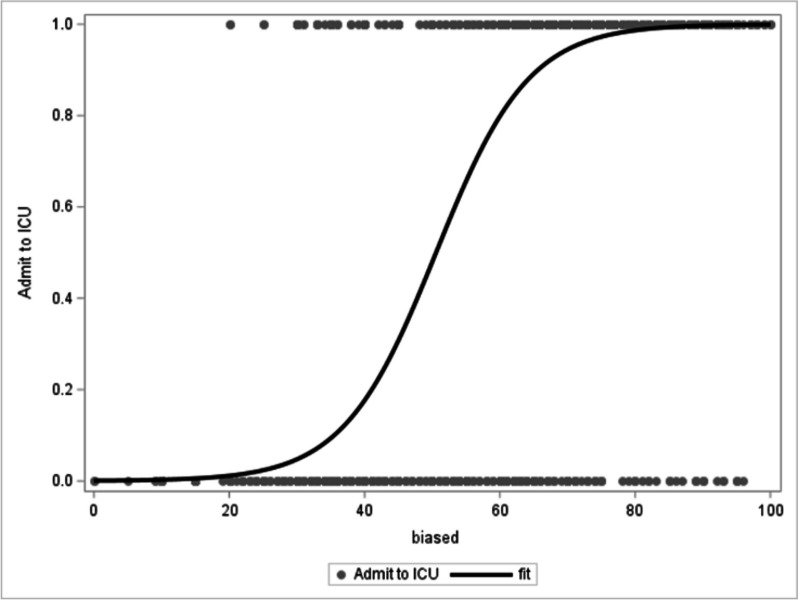
The probability of admission correlates with predicted survival. The survival rating after advice is shown on the *x*-axis, while the probability of admission (as estimated by the generalized linear mixed model) is displayed on the *y*-axis

The decision to admit a patient to the ICU was highly correlated with the rating after receiving advice (OR = 1.16, 95%CI (1.14–1.18), *p*-value < 0.0001) (Fig. 6). The generalized linear mixed model estimated 5%, 50% and 95% probabilities for ICU admission at a 30.4%, 50.6% and 70.8% rated survival probability, respectively. The strength of the association between rated survival probability and admission to ICU differed in qualification groups (*p*(interaction) < 0.0001) but did not change predictably according to the physician’s degree of experience (see Additional file 1: Fig. S4).

In the qualitative questionnaire completed at the beginning of the study session, participating physicians were first asked if and how they had learned about triage decision-making. They named a broad range of formal sources for learning about triage, including university and paramedic education, additional training offered by employers, scientific books, and journal articles, as well as various courses and conference talks. Informal sources included everyday interactions in clinical practice and in emergency response situations, as well as conversations with colleagues. Some participants noted that the formal education on triage they had received at university was insufficient, indicating that the other modes of learning about triage are crucial in preparing physicians for crisis situations.

Of the 32 participants, 8 (25%) stated that they either had not (yet) been actively involved in triage decision-making (*n* = 6) or did not have primary responsibility for making these decisions as there was a superior or team in place which took on this role (*n* = 2). The other 24 (75%) participants stated that they had made triage decisions in the past or were regularly involved in such decisions.

Although there were noticeable overlaps in the answers on making triage decisions and the specific criteria applied, there were different focal points, indicating that the applied criteria can and do vary between physicians. Several participants referred to official checklists and standardized clinical guidelines, as well as the patients’ frailty scores as the basis for triage decisions. Furthermore, several participants mentioned factors such as available resources and capacities, both in terms of human and technical resources (respirators, available ICU beds, etc.), when making triage decisions. Maximizing utility was identified as a triage goal by some participants, while others added that triage is applied not only as a means of deciding who will receive treatment but also to prioritize the patients most urgently needing care. Several physicians also stated that aspects of triage go beyond official checklists and are not “necessarily quantifiable”, as one participant phrased it, because clinical experience and “personal, experienced-based assessment” are also significant factors.

**Fig. 7:**
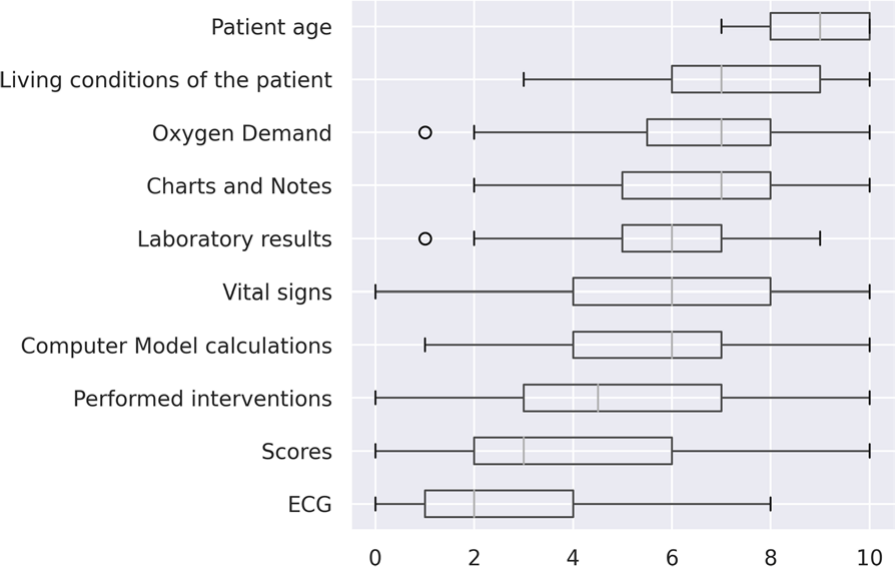
Influential factors in decision-making

**Fig. 8:**
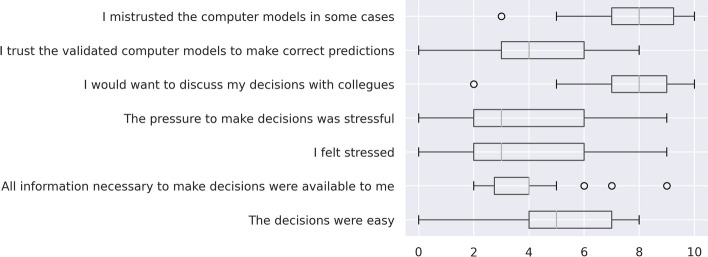
Final questionnaire

In the final questionnaire, completed at the end of the study session, participants were asked if they trusted the computer models in general; if they mistrusted the models in some cases; and to rate the impact the software models had on their decision. The median overall level of trust in the software was 4 out of 10 points (0 = no trust, 10 = absolute trust). Thirty out of 32 physicians responded to the question about distrust of the software in some cases by giving more than 5 points on a 0–10 scale. The physicians’ self-assessed value for overall trust in the software and its self-assessed influence on their decision-making correlated moderately with their median WoA (*r* = 0.56 and *r* = 0.81, respectively; *p*-value < 0.001 for both). An overview of the self-assessed influential factors and answers on the final questionnaire are given in Figs. 7 and 8. The most influential factor was patient age, followed by living situation (mean influence 8.9 (IQR 8–10) and 7.2 (IQR 6–9 out of 10). ECG (2.4, IQR 1–4) and calculated scores (3.8, IQR 2–6)) had the least influence on the rated survival probability. After all the experiments had been conducted, participants were asked to recall how much they trusted each system. Those who remembered rated the systems with an overall median of 5.5 (IQR 4–6.75 out of 10) points, and separately with a median of 4 (IQR 3.5–5.5 out of 10, no explanation) and 7 (IQR 5.5–7.5 out of 10, with explanation) points.

## Discussion

This investigator-initiated, prospective, experimental study showed that when tasked with predicting the survival probability of critically ill patients, physicians are significantly influenced by CDSS. However, contrary to our hypothesis, our data suggests no difference in weight-on-advice between the CDSS which explained its decision and the one that did not.

Ethical discussions about “intelligent machines” are as old as the idea of autonomous machines themselves. Isaac Asimov, a British writer, devised a set of rules known as *Asimov’s Laws*, which—when followed—should protect humanity from a robot uprising. The first rule states that a “robot may not injure a human being or, through inaction, allow a human being to come to harm” [19]. Consequently, adhering to these laws would prohibit CDSS from automatically denying treatment [20]. In accordance with this rule, the US Food and Drug Administration requires supervision of all machine-determined scorings by a qualified physician [21]. Nevertheless, in triaging patients, physicians rely heavily on scoring systems and increasingly on machine learning (ML) systems [20].

### Effect of explainability on WoA

Surprisingly, and contrary to our initial hypothesis, no difference could be found in the WoA based on explainability. Based on our data, two observations may provide some explanation for this unanticipated result.

Firstly, analysis of the post-experiment questionnaire revealed that physicians did in fact question certain parameters of the explanation provided by the CDSS. This may have led to increased scepticism of the specific CDSS and subsequent dismissal of its advice. The higher post hoc self-assessed trust in the computer models associated with a higher WoA supports this hypothesis. While our system simply provided a static, non-interactive explanation, Goldberg et al. described the importance of a structured decision-making process that focuses on interaction [22]. They argue that with the development of more sophisticated and reliable ML systems, users will become less critical of the recommended decisions and less prone to question them [22]. This claim of computer obedience is supported by experimental data [13], including the data from the present study. Goldberg et al. therefore suggest a process in which both the physician and the ML model are required to explain and “argue” their decision and question each other’s arguments. This has recently been termed a “clinical reasoning support system” [23]. It should be noted, however, that this kind of decision-making process is bound to require more time per decision than with conventional CDSS.

Secondly, our experiment intentionally put the participants under a high—but realistic—level of temporal stress, thus limiting the time available to question and understand the CDSS recommendation. Indeed, the authors of the “Barcelona Declaration for the proper development and usage of artificial intelligence in Europe” demand that artificial intelligence be able to explain its decision in a language the user is able to understand [24]. This emphasizes the need for improved, more intuitive presentation of the data informing the decision.

Interestingly, participants rated medical scores as the second to last influential factor in their decision-making process (mean 3.8/10, SD 2.5), while the computerized machine learning model was rated higher at a mean of 5.7/10 (SD 2.3). This further illustrates that subjects were unaware of the score-based nature of our CDSS.

### Taking advice

Our experiment was specifically designed to make participants feel overwhelmed and fatigued: physicians were presented with four binders of health records while, once again, being reminded of the set time limit. We hypothesized that due to the overwhelming and repetitive nature of the task, carried out in isolation, over the course of the experiment participants would exhibit decision fatigue. We hypothesized that WoA would increase and physicians would be more likely to accept the model’s recommendation [25–27].

Instead, no change in WoA could be observed suggesting that if decision fatigue occurred, it did not significantly influence WoA. The WoA remained remarkably constant over the course of the experiment, as well as over the course of the day (see Fig. 5). Additionally, the fact that 75% of participants have previously conducted or participated in triage processes may affect this invariability. This corresponds with comparable data presented by Zheng et al. [28]. The team analysed consultations and CT (computed tomography) orders over shifts in the emergency department and observed no decrease in consultations and admissions, concluding that decision fatigue does not occur in 8-h shifts at their emergency department [28]. By contrast, Häusser et al. showed that sleep deprivation might result in a significant increase in WoA in estimation experiments [18]. Furthermore, they showed that the competency of the advisor was associated with a higher WoA [18]. This current research suggests that WoA is not a mere constant but rather a complex function of various experimental parameters [18, 29]. For instance, the extent of the difference between the original rating and the subsequent advice given by the CDSS has also been shown to affect WoA [29]. Despite this, studies show that when taking advice, regardless of the experiment, the mean WoA is approximately 30%, slightly lower than in our observations [17, 30]. In conclusion, the constancy of WoA throughout the duration of our experiment might be rooted in the comparative briefness of our experiment together with the perceived incompetence of our supposed CDSS.

Soll et al. [31] and Lees et al. [32] have raised concerns that calculating the mean of the WoA measurements may hide the true bimodal distribution of WoA. Indeed, the premise of WoA bimodality, computed using Hartigan’s dip test [33], holds in our dataset (*p*-value < 0.001) [34]. In contrast to the observation of Lees et al. [32], we did not observe peaks at the ends of the WoA spectrum (0 and 1); instead, peaks were located at 0% (no change following advice, 30% of data points) while the remaining data points had a median of 50% (averaging the own rating with the CDSS rating).

Interestingly, our data suggests that physicians were aware of being significantly influenced by the CDSS. A higher WoA was associated with a higher self-assessed impact of the CDSS on the physician’s decision, in addition to greater perceived trust in the CDSS.

To the best of our knowledge, this is the first study to examine the physician–CDSS interaction in the situation of repetitive tasks requiring serial decision-making. However, the important limitations of our study need to be addressed. Firstly, while we specifically designed the experiment to resemble a plausible real-world situation, the experimental nature and its associated limitations of the study remain. Secondly, explainability is a broad term that does not describe a well-defined property of CDSS. Instead, explainability may take many forms. Third, a highly selected patient cohort of critically ill patients served as a basis for our study. Thus, while we have presented important learnings in the field of CDSS and human interaction, the ability to generalize our results may be limited.

## Conclusions

An overall high weight-on-advice (WoA) of 34% could be observed in our data, indicating that on average the physicians’ final rating moved 34% of the distance between the initial rating and the systems’ advice. At the same time, the median trust in the computer models was low and awarded only 4 points on a 10-point scale.

In light of its fundamental consequences, the process of ICU triage must be based on a robust decision-making process. Emerging technologies allow this process to be facilitated using ML-based CDSS systems. However, the implications of consulting CDSS in this situation are neither well understood and nor have they been examined to date. Further research is urgently needed to examine how CDSS interact with the decision-making processes made by healthcare professionals in triage situations. Newer techniques such as clinical reasoning systems could potentially improve clinician–CDSS interaction, thereby improving patient safety.

### Supplementary Information

**Additional file 1: Fig. S1.** Patient chart cover page. **Fig. S2.** Quality of randomization. **Fig. S3.** Weight-of-advice by physician and explanation. **Fig. S4.** Probability of admission based on rating after advice and level of qualification.

## Data Availability

The datasets used and/or analysed during the current study are available from the corresponding author upon reasonable request.

## Abbreviations

CDSS: Clinical decision support system
ICU: Intensive care unit
IQR: Interquartile range
ML: Machine learning
OR: Odds ratio
SD: Standard deviation
WoA: Weight-on-advice
