# Supplementary material for: The influence of explainable vs non-explainable clinical decision support systems on rapid triage decisions: a mixed methods study
Source: BMC Med. 2023 Sep 19;21:359. doi: 10.1186/s12916-023-03068-2 (PMC10510231; doi:10.1186/s12916-023-03068-2)
Supplement: Supplementary file 1 — Additional file 1: Fig. S1. Patient chart cover page. Fig. S2. Quality of randomization. Fig. S3. Weight-of-advice by physician and explanation. Fig. S4. Probability of admission based on rating after advice and level of qualification. [file 12916_2023_3068_MOESM1_ESM.docx]

Additional File 1 to “The Influence of Explainable vs. Non-Explainable Clinical Decision Support Systems on Rapid Triage Decisions: A Mixed Methods Study”

Contents:

- Figure S1: **Patient Chart Cover Page**
- Figure S2: **Quality of Randomization**
- Figure S3: **Weight-of-Advice by physician and explanation**
- Figure S4: **Probability of admission based on rating after advice and level of qualification**

# Figure S1


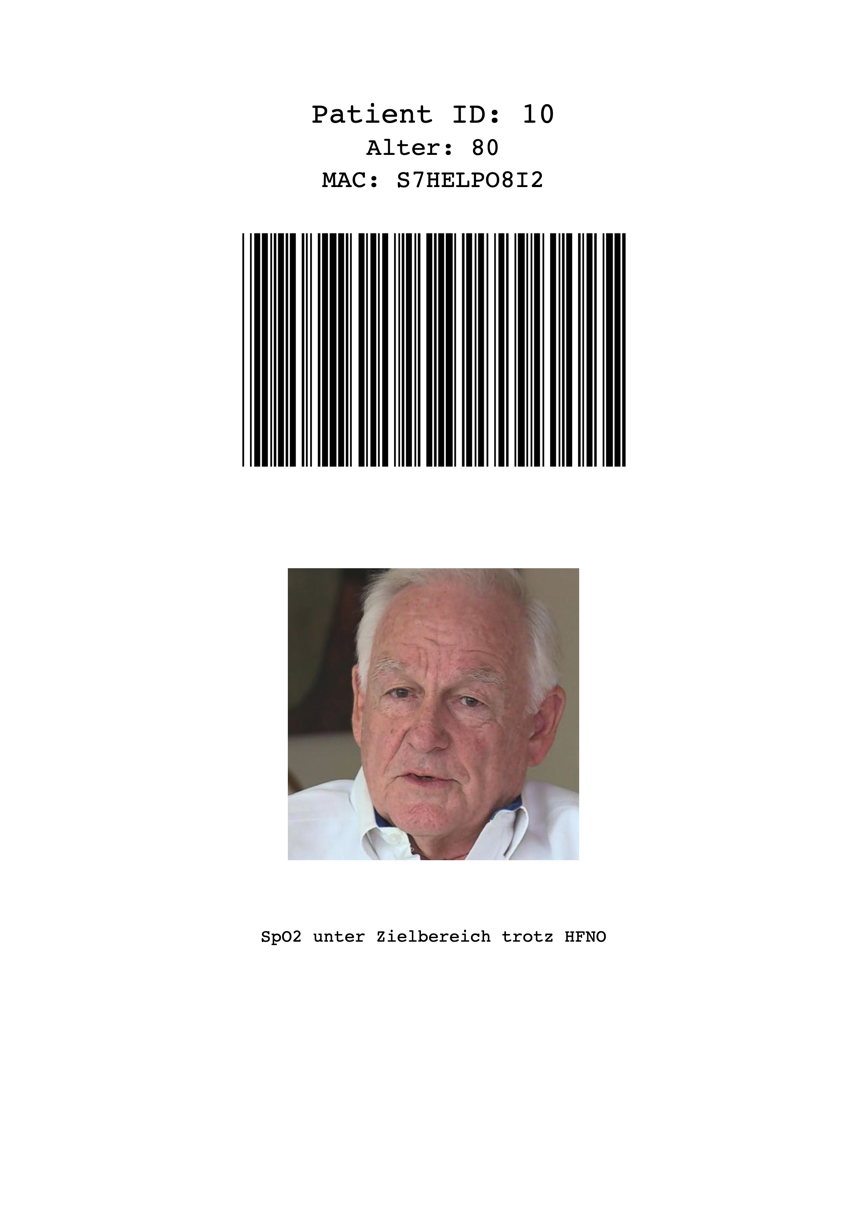


**Patient Chart Cover Page**

This image shows a cover of a patient chart including ID, age, barcode, image, and indication for ICU therapy. All patient images were obtained from <https://this-person-does-not-exist.com/en>. The text under the image translates as “SpO2 under target range despite of HFNO”.

SpO2: peripheral Oxygen saturation; HFNO: High-flow Nasal Oxygen

# Supplemental Figure S2


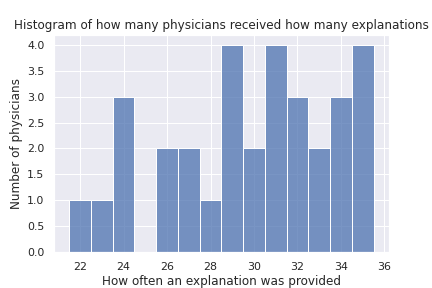


**Quality of Randomization**

This chart shows the quality of randomization. On the x-axis the number of patients an individual physician received an explanation on is shown (out of 59). The y-axis shows the number of physicians with this number of explanations.

Overall an explanation was provided for 955 patients while no explanation was provided for 933 patients.

# Figure S3


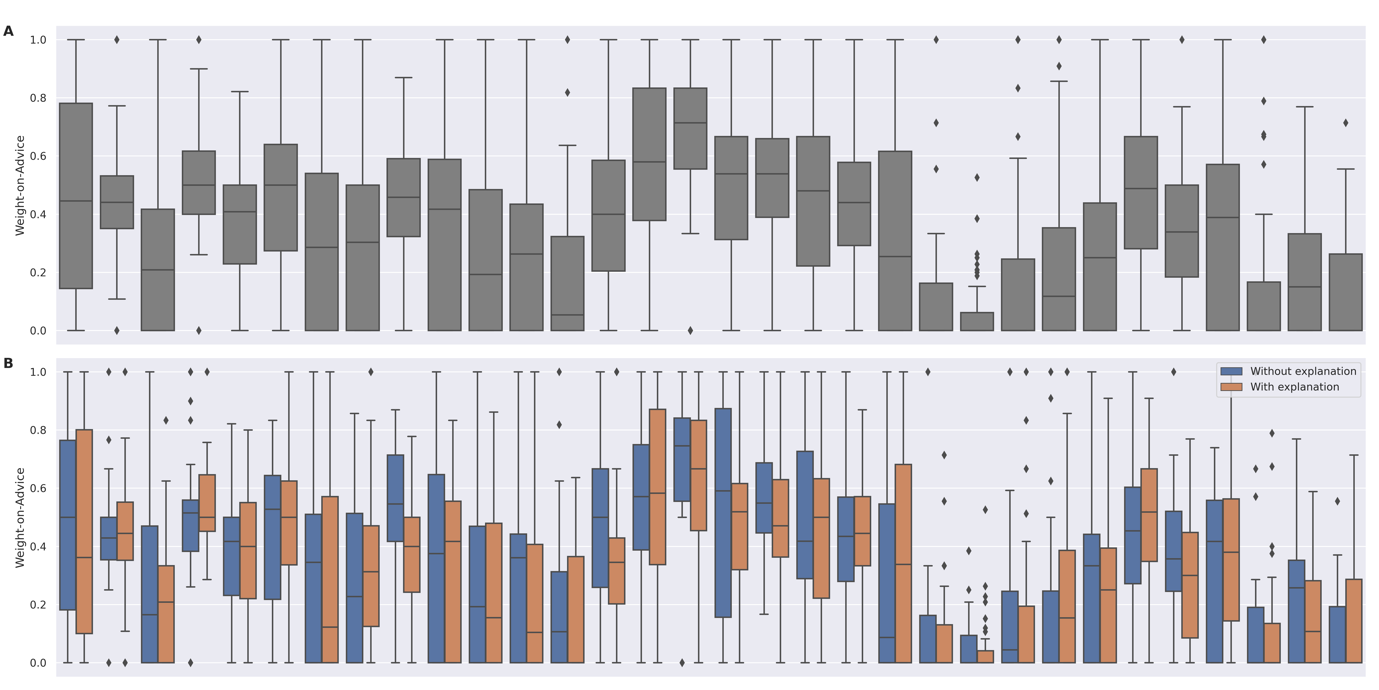


**Weight-of-Advice by physician and explanation**

Weight-of-Advice grouped by physician (Panel A) and grouped by physician and whether an explanation was provided (Panel B).

# Figure S4

**
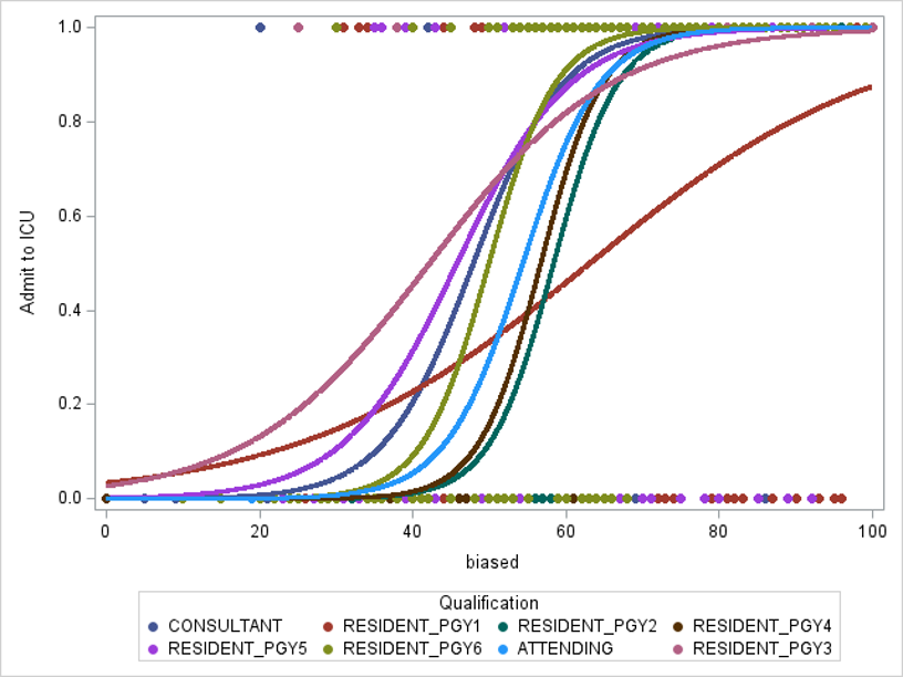
**

**Probability of admission based on rating after advice and level of qualification**

The x-axis shows the survival probability given after receiving advice. The y-axis shows ICU admittance.

PGY: Postgraduate Year. Austrian residency lasts for six years.
